# Identifying Nontraditional Epidemic Disease Risk Factors Associated with Major Health Events from World Health Organization and World Bank Open Data

**DOI:** 10.4269/ajtmh.20-1318

**Published:** 2021-08-30

**Authors:** Roberta Lugo-Robles, Eric C. Garges, Cara H. Olsen, David M. Brett-Major

**Affiliations:** ^1^ Department of Preventive Medicine and Biostatics, Uniformed Services University, Bethesda, Maryland;; ^2^ Henry M. Jackson Foundation, Bethesda, Maryland;; ^3^ College of Public Health, University of Nebraska Medical Center, Omaha, Nebraska

## Abstract

Health events emerge from host, community, environment, and pathogen factors—forecasting epidemics is a complex task. We describe an exploratory analysis to identify economic risk factors that could aid epidemic risk assessment. A line list was constructed using the World Health Organization Disease Outbreak News (2016–2018) and economic indicators from the World Bank. Poisson regression employing forward imputations was used to establish relationships with the frequency with which countries reported public health events. Economic indicators demonstrated strong performance appropriate for further assessment in surveillance programming. In our analysis, three economic indicators were significantly associated to event reporting: how much the country’s urban population changed, its average forest area, and a novel economic indicator we developed that assessed how much the gross domestic product changed per capita. Other economic indicators performed less well: changes in total, female, urban, and rural population sizes; population density; net migration; change in per cent forest area; total forest area; and another novel indicator, change in percent of trade as a fraction of the total economy. We then undertook a further analysis of the start of the current COVID-19 pandemic that revealed similar associations, but confounding by global disease burden is likely. Continued development of forecasting approaches capturing information relevant to whole-of-society factors (e.g., economic factors as assessed in our study) could improve the risk management process through earlier hazard identification and inform strategic decision processes in multisectoral strategies to preventing, detecting, and responding to pandemic-threat events.

## INTRODUCTION

The challenge of employing infectious disease outbreak data usefully to help those managing an emergency and the need to purposefully develop models oriented to decision-makers and in a context of being connected to management, is increasingly recognized.^1^ Monitoring population health, which includes demographic and health surveillance and epidemiological studies, can generate valuable data that can be used in health prediction models.^2^ There are several barriers to this. Among them are availability of validated data before an emergency to proof the model and during an event to provide actionable information. Such efforts focus on early warning of an event or its trajectory. They often are anchored on specific characteristics of how an outbreak pathogen behaves. This may result in clumsy applications of information oriented in that way to a community-based perspective of what must be done to prevent and mitigate risks. Here, we attack these challenges from the flank, pursuing in a pilot analysis economic indicators at the population level that may assist prediction in broad strokes across many pathogens, seeking to demonstrate potential utility of openly available information from both health and nonhealth interagencies, and exploring triggers meriting prevention and early mitigation efforts.

The WHO Disease Outbreak News (DON) is a major conduit for information sharing relevant to state party obligations under the International Health Regulations. Last published in 2007, the International Health Regulations includes an assessment and notification tool to report events that may constitute a public health emergency of international concern.^3^ The outputs from this risk identification tool are translated into brief reports for the DON. Each WHO Member State is meant to report events in accordance with these regulations.^3^

**Figure 1.**
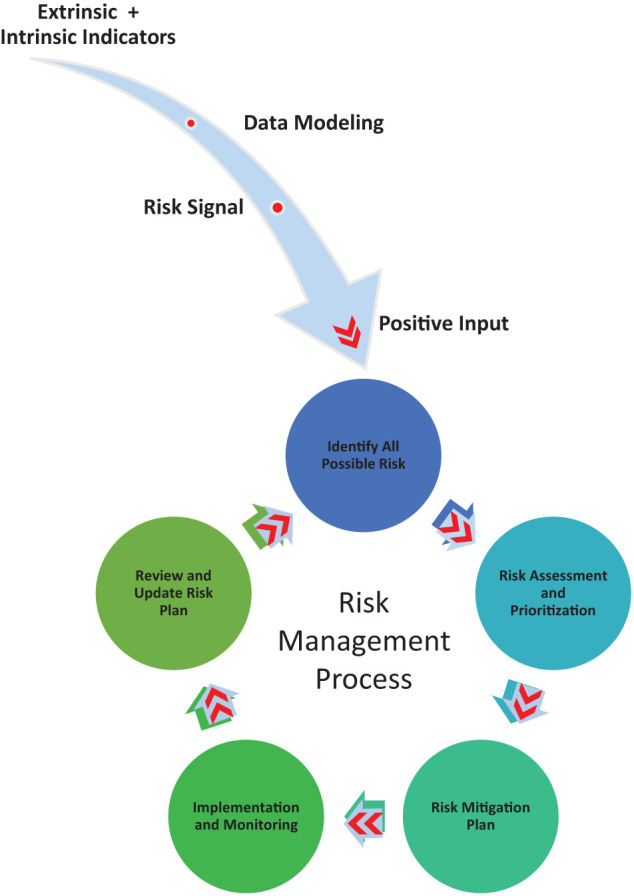
Risk signal identification and input effect on the risk management process. This figure appears in color at www.ajtmh.org.

We aimed to 1) describe reported outbreak events impactful to communities via the WHO DON from 2016 to 2018, 2) identify and explore major economic indicators at the population level potentially related to these events, and, 3) generate hypotheses regarding associations between economic factors and events for the purpose of identifying triggers for enhanced surveillance, other health system strengthening, or holistic community interventions that might later be investigated for either early risk mitigation or prevention of pandemic-threat events. Detecting early risk signals boosts the risk management process (Figure 1) through longer lead time for risk identification and characterization.

## METHODS

Information contained in WHO DON from 2016 to 2018 was reviewed and used to construct a line listing. Each row of the line listing identified a single outbreak (event) in a single country. Multiple reports corresponding to the same outbreak (or event) were summarized within the same row. Outbreak information such as demographics and epidemiological indicators were recorded, as available. In order for a country to be included in analyses, it had to have reported a health event to WHO resulting in a WHO Disease Outbreak News release. Ninety-six countries met this criterion.

Economic indicators were sought to represent extrinsic factors, that is, nonbiological factors that could contribute to the risk of a health event regardless of the pathogen involved. As outbreaks spread and spatiotemporally separated waves become entangled with human mobility, behavioral changes, pathogen evolution, and other factors, the power of prediction models decrease despite increasing time-series lengths.^4^ This makes the prediction of an event complex. We limited our study to evaluate each of a specific number of potentially predictive associations between economic indicator factors and frequency of reported health events. We used World Bank’s World Development Indicators, which are internationally comparable statistics about global development selected as community-centric risk factors that are routinely assessed and could inform a risk management and prioritization process. We surveyed communicable disease burden modeling efforts in the literature and selected eight of these factors (MEDLINE search terms: emerging infectious diseases prediction, health events forecast, and extrinsic health factors) after consultation with an economist familiar with World Bank metrics. Our interest in signal detection (something has changed and so a new risk management action may be appropriate) influenced selection. The selected factors were Population Change, Female Population Change, % Forest Area Change, Forest area square kilometers, Population Density, Net Migration, Urban Population change, and Rural Population change. We also developed and incorporated two novel indicators that we calculated from the World Bank indicators—gross domestic product (GDP) change per capita and trade as a % of GDP change—testing a hypothesis that economic change in either direction is associated with risk as it relates with how people interact within a community with both each other and their environment. Change was determined for economic indicators by calculating an average delta (Δ) as the difference between averages per annum baseline values (2006–2008) and average current per annum values (2016–2018).

### Data management.

Data were aggregated using country as a grouping variable. Events’ frequency (dependent variable) was summed and grouped per country. All the countries reported at least one event within the timeframe analyzed. Independent variables related to economics, population level, and environment were collected at baseline and current years. Baseline values were considered 10 years before (2006–2008) the DON reports included in the line list (2016–2018). Independent indicators were extracted from the World Bank open data repository. This database contains 1,600 time series indicators for 217 economies and more than 40 country groups from the past 50 years.^5^

### Statistical analysis.

Descriptive statistics were used to describe the health events in terms of age, sex, case count, death count and other variables. Nonparametric statistics were applied to the dataset. Bivariate analyses exploring associations included Spearman’s rank correlation coefficient and Mann–Whitney *U* tests. Country comparisons to identify a potential confounding factor related to economic reporting behaviors relevant to achieving aid were performed. Data related to global health security funding commitments from 2014 to 2019 for the three countries in our data set with the most and least absolute GDP per capita change was retrieved and compared using the Georgetown Infectious Disease Atlas.^6^

Poisson regression models employing forward imputations were used to establish relationships and predict values over the dependent variable, particularly to characterize the performance of the new surveillance indicators in the model and validity. Statistical model fitting was assessed by omnibus test (*P* < 0.001) and the goodness of fit. Over-dispersion was evaluated by Pearson chi-square value from the goodness of fit. All statistics were considered significant at a *P* value of 0.05. A Monte Carlo simulation was used employing the Poisson regression results. All input variables were fitted before running simulations to include tests for interaction.

## RESULTS

**Figure 2.**
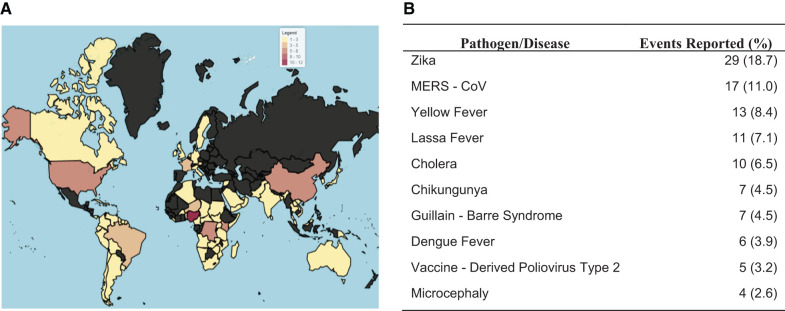
Overview of the reported health events in the WHO Disease Outbreak News (2016–2018). Economic indicators were tested against the frequency with which a particular country reported a health event meriting a WHO Disease Outbreak News release, a mechanism of International Heath Regulations (2005) compliance. This figure provides an overview of the distribution and dominant event types that constituted that dataset. (**A**) Geographic heat map displaying the frequency of health events reported by country. (**B**) Frequency of the most common of the pathogens/diseases reported. This figure appears in color at www.ajtmh.org.

From 2016 to 2018, 96 countries reported to WHO through the DON the amount of 155 health events from 29 pathogens (Figure 2A). The most common pathogens/disease events reported are displayed in Figure 2B. Zika outbreaks represented 18.7% of the health events reported, followed by MERS-CoV (11.0%) and yellow fever (8.4%). A dengue virus event was the largest reported, with more than 94,000 cases, followed by cholera with 27,978 cases. The demographic characteristics of the countries with more health events and least health events reported are depicted on Table 1. We observed no overall trend when comparing demographic indicators between countries with the highest number of reported health events against those with the lowest number reported (a single report).

**Table 1:** Countries’ Demographic Characteristics for 2018

Country	Health events	Population	Mortality rate, under 5 (per 1,000 live births)	Birth rate, crude (per 1,000 people)	Death rate, crude (per 1,000 people)
Nigeria	12	195,874,740	120	38	12
Democratic Republic of Congo	7	84,068,091	88	41	9
China	6	1,392,730,000	9	11	7
United States	6	326,687,501	7	12	9
France	5	66,977,107	4	11	9
Cuba	1	11,338,138	4	10	9
Dominica	1	71,625	36	12	8
Guyana	1	779,004	30	20	7
United Kingdom	1	66,460,344	4	11	9
Malaysia	1	31,528,585	8	17	5

The top five and the least five countries reporting health events to WHO Disease Outbreak News.

Economic indicators tested against reported health event frequency are described in Table 2. Total population and GDP per capita had the greatest magnitude of change. The least changing indicator was female population with an overall mean change of –0.17%. In indicator validation, bivariate Poisson test results showed that the economic indicators GDP change per capita, urban population change, and current average forest area were significantly associated with health event frequency (*P* = 0.001, 0.004, and < 0.001, respectively). Net migration change was the least related indicator (*P* = 0.838).

**Table 2:** Description of the economic factors evaluated

Economic factors	2006–2008	2016–2018	Mean change
	Mean (SD)	Mean (SD)	
GDP per capita (US 100 dollars)	111.77 (167.82)	120.73 (167.02)	8.96*
Population (million people)	59.28 (191.57)	66.43 (208.08)	7.14*
Urban population (%)	54.66 (24.15)	57.96 (23.84)	3.30*
Female population (%)	49.43 (3.94)	49.26 (4.52)	−0.17
Population density (people per square kilometer of land area)	186.32 (266.45)	215.22 (324.09)	28.90*
Net migration†	6,428.69 (993,765.53)	−15,462.46 (744,692.31)	−21,127.50
Forest Area (million sq/km)	32.32 (25.31)	32.10 (25.47)	−0.39
Trade (%)	90.06 (78.08)	76.38 (43.10)	−16.60*
Age 65 and older (years)	6.26 (4.59)	7.42 (5.70)	1.16*

*N *= 96 countries from 2016 to 2018.

* Paired *t*-test significant at *P* < 0.05.

† Net migration data was available from 2007 and 2017.

After testing all covariates (nine in total) against the dependent variable: health event frequency, multiple models were constructed for the purpose of exploring covariate associations. Ultimately, three variables in the Poisson analyses were associated significantly with event frequency (population urban change, GDP change per capita, and the average forest area [2016–2018 current average]) and a fourth variable (percent of trade change) that was not statistically significant alone in the model interacted with the other covariates (Table 3). This suggested that an increase in the GDP change increases the likelihood of an event by 1% (*P* = 0.00), increase in urban population change raises the likelihood by 7.2% (*P* = 0.011), and an increase in average forest area (square meter) increases the likelihood by 27.9% (*P* = 0.002). The goodness-of-fit Pearson chi-square was 0.850, suggesting a good fit of our data to a Poisson distribution in the regression, with no apparent impact from covariate interactions. The likelihood ratio chi-square results showed GDP change per capita, population urban change, and average forest area have a discernible effect (*P* < 0.05).

**Table 3:** Poisson regression model parameters estimates for health event frequency

Explanatory variable	Rate ratio	95% CI for rate ratio		Significance level (*P* value)
(Intercept)	1.024	0.740	1.418	0.885
GPD change per capita (US 100 dollars)	1.010	1.006	1.015	< 0.001*
Population urban change (%)	1.072	1.016	1.132	0.011*
Average forest area sq. meter (million sq/km)	1.279	1.095	1.494	0.002*
Trade percent change (%)	1.000	0.997	1.003	0.932

CI = confidence interval. Dependent variable: event frequency. *N* = 96 countries from 2016 to 2018.

* *P* value < 0.05.

**Figure 3.**
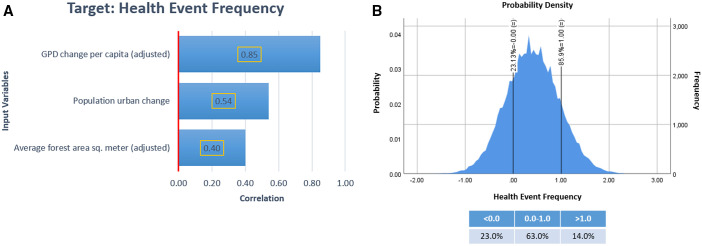
Monte Carlo simulation output using Poisson equation regression model. (**A**) Correlation tornado chart for input variables: gross domestic product change per capita, population change and average forest area. (**B**) Probability density chart of health event frequency; reference probability values were placed at 0.0 and 1.0 events. This figure appears in color at www.ajtmh.org.

Monte Carlo simulations were used to further assess the resilience of observed associations, showing strong correlations with GDP change per capita, population urban change, and average forest area in Monte Carlo simulations. A total of 71,075 cases were simulated using Monte Carlo method to meet the confidence interval of the mean of the target variables (health events), at the 95% confidence level. The tornado chart (Figure 3A) shows a strong Pearson correlation between health events and GDP change per capita (adjusted) of 0.85 and moderate correlations for the input variables population urban change and current average forest area (adjusted) with correlation coefficients of 0.54 and 0.40, respectively. The probability density chart (Figure 3B) displayed the distribution of the target variable (health events frequency) simulated by the Monte Carlo method. Results show a probability of 14% to have more than two health events and a probability of 87.9% to have at least one health event with our Poisson analysis indicators.

A confounding analysis related to economic reporting behaviors was performed. It showed Venezuela, Nigeria, and the United States with the greatest GDP change and Haiti, Bahrain, and Pakistan with the least change. Among the low- and middle-income countries, global health security funds commitments were comparable for size with the exception of Venezuela; however, given the political distance between the major global health security donors and that country during the time period, this was not surprising. After evaluating comparisons among countries, an economic reporting bias was not found.

In light of the ongoing novel coronavirus disease (COVID-19) public health emergency of international concern, we undertook an additional exploratory analysis of COVID-19 cases in the WHO DON through 30 April reported in the countries included in our dataset, seeking to represent initial case rises rather than the pervasive and persistent aspects of that health emergency. A correlation analysis showed GDP change per capita (*P* = 0.024) and average forest area (*P* < 0.001) linkages to COVID-19 case counts. This is further supported with linear regression demonstrating that GDP change per capita, average forest area, and urban population change influence the outcome variable—in this case, COVID-19 cases reported (*P* < 0.001). An *R* = 0.5 suggests moderate correlation, and a 32% variance proportion in the dependent variable (COVID-19 reported cases, *R*^2^) can be predicted from these same three variable.

## DISCUSSION

Research that pushes how surveillance and related activities happen is important.^7^ Doing so can be challenging for many reasons. Medical intelligence and surveillance are increasingly multisectoral in nature resulting in many stakeholders that both compete and collaborate. Nonetheless, opportunities for enhancing public health practice exist in terms of increased scientific rigor, outcomes-focused research, and health informatics.^7^ We sought a novel application of unconventional data for these purposes, selecting the WHO DON because the events matter to communities, and the World Bank economic development indicators as they reflect broad aspects of community wellness. We applied them holistically in a way that mitigates their internal validity issues.

Our work here only includes WHO countries that reported events during 2016–2018, resting on premises that past experiences are related to future experiences and that these countries offer lessons for those that have not yet reported such events, even if they may have experienced analogous ones. We evaluated nine extrinsic factors; four of these were significantly related to frequency of a reported emerging infectious diseases event, with the average forest area square meters being the extrinsic factor with the highest effect in the Poisson analysis. Our simulation showed that there is an 87% probability that a country will experience a health event during a 3-year observation period if it has undergone significant GDP, trade, and population changes in the previous 10 years while having a large forest area. This suggests that extrinsic factors related to a health event must be incorporated, or a tiered approach adopted, to have a fuller picture of community and patient vulnerabilities for an event.

Previous studies have shown that extrinsic factors are associated with health, such as green space area, population density, wealth, education and others.^8^^–^^11^ Our results showed an association between forest area and health event. More forest area available was associated with an increased risk of reported emerging infectious disease events. Changes in land cover and land use, including forest area change (particularly deforestation and forest fragmentation), urbanization (which is included here as population urban change), and agricultural intensification are major factors contributing to the surge in infectious diseases.^12^ A recent study sought to forecast the next forest-based emerging infectious disease and concluded that southern and eastern forests around Freetown in Sierra Leone, the forest region around Douala in Cameroon, or the southern forest region in Nigeria were potential upcoming originating centers of emerging infectious disease.^13^ These regions have extensive forest areas, consistent with our assessment, presumably from increased opportunities for zoonotic cross-over events. Exposure time is a dominant feature of risk.

Urban population change had a moderate association with health events when tested with other extrinsic factors. In 2014, the WHO asserted that urban areas held 54% of the total global population.^14^ This ratio is expected to increase from 55% in 2018 (some 4.2 billion people) to 68% by 2050.^15^ Our findings suggest that undergoing an urban population change increases risk of a health event. Whether this is better explained by forest incursion from urbanization, human consolidation and so increased human-to-human exposure time, aspects of domestic travel and draw from more wild fringe areas prone to sentinel zoonotic events, or other root causes is less clear. Incorporation of more local market and migration factors could assist in making these distinctions. Regardless, with rapid global urbanization, understanding relationships between the changing urban environment and human health is vital. Urban environments play an indispensable role in influencing human health and well-being.^16^

Human population density has been recognized as a putative driver of emerging infectious diseases.^17^ Human behavioral changes regarding movement and urbanization are thought to contribute to this.^18^ Megacities may serve as incubators for new epidemics and zoonotic diseases of rapid spread.^19^ Sporadic encounters between wildlife and humans in urban areas may become more frequent in periurban settings, resulting in greater exposures to parasites, dengue virus, cholera, tuberculosis, Lyme, and other threats.^17^^,^^18^^,^^20^^–^^24^

Our statistically strongest association rested on whether the gross domestic product of a country had undergone change in the intervening decade. Studies have shown associations between GDP and health outcomes, as well as increased health care expenditures and GDP growth.^25^^,^^26^ We specifically tested whether a changing GDP changed risk for a health event. This bore from our suspicion that whether increasing or decreasing, a changing GDP indicated a condition under which communities must adapt in ways that seek new markets and ways of instigating commerce, sporadic and sustained forest contact, evolving interactions with urban areas, and changing patterns of interactions between persons. Indeed, our results demonstrate that GDP change per capita in either direction increases the probability of an emerging infectious disease event. This suggests that all countries are vulnerable to this effect regardless of baseline GDP.

Our work focused on indicator development and did not seek to establish a prediction or forecasting model. There are, however, relevant new initiatives aimed at how risk models for infectious diseases are developed, such as the Epidemic Prediction Initiative from the Centers for Disease Control and Prevention.^27^ Although this initiative is meant to facilitate open forecasting projects toward public health decision-making, they are targeted to explore specific pathogens, diseases, or vectors (e.g., dengue, influenza, *Aedes aegypti*, and *Aedes albopictus*).^27^ Our approach may be particularly useful for whole-of-society planning and operational health emergency risk management processes, such as that called upon in the Joint External Evaluation monitoring progress in country attainment of International Health Regulations 2005 capacities, as well as other capability development and exercise initiatives.^28^

The main advantages of our approach are simplicity; ready hypothesis generation from existing open source, long-lived data mechanisms; and the ability to be adapted to specific epidemiological scenarios, including contextual layering on current pathogen-specific prediction models. Employing nonhealth factors may improve surveillance and modeling practice in a variety of ways, including model accuracy when predicting risks from pandemic threats. It offers readily observable triggers for initiating targeted risk assessments and planning, and it may improve event-based surveillance.^29^

Our exploration into these effects impact on COVID-19 risk were limited. However, these results suggest that COVID-19 disease dynamics (e.g., outbreak magnitude, cases, death toll) when reported could be affected by the economic indicators identified in our findings. The analysis may have been heavily confounded by the pervasiveness of COVID-19 in the pandemic setting.

Our main limitations revolve around data collection in low-resource settings and reporting in politically associated systems. Variability existed in how data were reported in the WHO DON. Demographic characteristics and epidemiological indicators were sometimes nonspecific or missing. Under-reporting of cases occurred. For example, although Ebola virus disease, influenza, and Zika events were reported when the outbreak started in the country, subsequent reports of the outbreaks were omitted or contained limited information. Data extraction was made from WHO DON reports; therefore all the countries incorporated in the analysis have at least one event reported. On one hand, this introduces bias and limits the applicability of the findings to the nonreporting countries on the studied timeframe; on the other hand, countries without a report were relatively few, and the presence of at least one report indicates that the country will and has the mechanism to report. Although our confounding analysis regarding country wealth, funding, and reporting behaviors yielded reassuring results, confounding may still be present. Additionally, we did not incorporate community and governmental action in preparedness and response in our assessments of event likelihood; however, the persistence of effect across resource levels suggest that although such analysis would be valuable, it would be complementary rather than obviating.

This is the first step in a path of work. We are interested in exploring and eventually characterizing how nonhealth indicators might be incorporated into the ways that surveillance across the event- and indicator-based spectrum are conducted. We perceive the value of economic indicators as being fundamentally connected to community-centered outcomes and purposefully in our construct oriented the tests for associations in that way, including why we chose the outbreak data sets that we did. Although not our focus, eventually greater incorporation of such indicators in more conventional health system modeling should be tested. Our approach, in contrast, is to test the utility of such economic indicators as triggers for deeper whole-of-society action on health emergency prevention, readiness, and resiliency and understanding the immediacy of threats.

## CONCLUSION

Our exploratory analysis demonstrates that economic factors are associated with whether a country experiences a significant health event from an emerging or reemerging infectious disease, based on major health events (e.g., infectious diseases outbreaks) reported by WHO member states between 2016 and 2018. We developed a novel indicator, GDP change per capita, which had the statistically strongest association in our analyses. Established economic indicators of urban population and forest area also were associated with higher frequencies of health event reporting. Further exploration of dynamic economic factors as tools in prediction of such events anchored in community-centered outcomes is merited. Even now, indicators representing these factors as well as from other disciplines may be valuable for use case applications to contextualize whole-of-society threat-planning processes and medical intelligence, leading to public health decision-making for priority surveillance and other health and nonhealth investments.
